# GPrimer: a fast GPU-based pipeline for primer design for qPCR experiments

**DOI:** 10.1186/s12859-021-04133-4

**Published:** 2021-04-29

**Authors:** Jeongmin Bae, Hajin Jeon, Min-Soo Kim

**Affiliations:** 1 Korea Advanced Institute of Science and Technology, KAIST, 291, Daehak-ro, Yuseong-gu, 34141 Daejeon, South Korea; 2 Department of Information and Communication Engineering, DGIST, 333, Techno jungang-daero, Hyeonpung-eup, Dalseong-gun, 42988 Daegu, South Korea

**Keywords:** Primer design, GPU computing, Sequence analysis

## Abstract

**Background:**

Design of valid high-quality primers is essential for qPCR experiments. MRPrimer is a powerful pipeline based on MapReduce that combines both primer design for target sequences and homology tests on off-target sequences. It takes an entire sequence DB as input and returns all feasible and valid primer pairs existing in the DB. Due to the effectiveness of primers designed by MRPrimer in qPCR analysis, it has been widely used for developing many online design tools and building primer databases. However, the computational speed of MRPrimer is too slow to deal with the sizes of sequence DBs growing exponentially and thus must be improved.

**Results:**

We develop a fast GPU-based pipeline for primer design (GPrimer) that takes the same input and returns the same output with MRPrimer. MRPrimer consists of a total of seven MapReduce steps, among which two steps are very time-consuming. GPrimer significantly improves the speed of those two steps by exploiting the computational power of GPUs. In particular, it designs data structures for coalesced memory access in GPU and workload balancing among GPU threads and copies the data structures between main memory and GPU memory in a streaming fashion. For human RefSeq DB, GPrimer achieves a speedup of 57 times for the entire steps and a speedup of 557 times for the most time-consuming step using a single machine of 4 GPUs, compared with MRPrimer running on a cluster of six machines.

**Conclusions:**

We propose a GPU-based pipeline for primer design that takes an entire sequence DB as input and returns all feasible and valid primer pairs existing in the DB at once without an additional step using BLAST-like tools. The software is available at https://github.com/qhtjrmin/GPrimer.git.

## Background

Quantitative polymerase chain reaction (qPCR) (also known as real-time PCR) is a standard technique widely used for detecting the mass amplification of specific DNA molecule in real-time. Its applications include virus detection [1], genetically modified organism (GMO) detection [2], pathogen discovery [3], and validation of changes in expression of interested genes [4]. For best results in qPCR experiments, design of high quality primers is more important than anything. MRPrimer [5] is a MapReduce-based powerful pipeline that combines both primer design for target sequences and homology tests on off-target sequences. In general, high quality primers should not only satisfy single and pair filtering constraints (e.g., primer length, melting temperature, GC content) to amplify target sequence(s) properly, but also pass homology tests not to amplify off-target sequences. Different from the conventional methods that do homology tests as an additional step using BLAST-like tools, MRPrimer takes an entire sequence DB and the filtering constraints as input and returns all feasible and valid primer pairs existing in the DB without an additional step using BLAST-like tools. In terms of finding all feasible and valid primer pairs existing in the DB at once, MRPrimer is quite different from the conventional primer design tools such as Primer3Plus [6] and PrimerBlast [7], which find only primer pairs existing in a single sequence. Due to the effectiveness of primers designed by MRPrimer in qPCR analysis, many tools including MRPrimerW [8], MRPrimerV [1] and MRPrimerW2 [9] have been developed based on MRPrimer and widely used. MRPrimerW is a web-based design tool that allows user to easily obtain the best set of primer pairs and TaqMan probes for batch qPCR experiments that should satisfy the set of stringent and uniform constraints as well as pass homology tests. MRPrimerV is a pipeline that can build a database of primers for detection of 1818 RNA viruses by taking both virus sequence DB and host (e.g., human, camel) sequence DB as input. MRPrimerW2 is an enhanced web-based design tool that supports exon spanning design, avoiding SNP sites, input FASTA sequences, and multi-target designing.

Although the MRPrimer pipeline is used as a core engine for the above primer design tools, it has a major drawback that its computational speed is too slow to deal with large-scale sequence DBs. MRPrimer is a MapReduce-based pipeline, and there are also many other MapReduce-based pipelines for sequence data analysis including CloudBurst [10], GATK [11], DistMap [12], MegaSeq [13], Halvade [14], Halvade-RNA [15], Rail-RNA [16], MarDRe [17], MEC [18] and KCH [19]. Originally, MRPrimer has been proposed to be based on the MapReduce framework running on a cluster of machines for fast and scalable data processing. Nevertheless, as the sizes of sequence DBs are growing exponentially due to the advancement of the sequencing techniques, MRPrimer may take too long time to design all primer pairs from the sequence DBs. For instance, in the study of MRPrimerV [1], MRPrimer takes more than two weeks for 101,684 human gene sequences even using 40 nodes of a supercomputer (Rank #454 in TOP500 Supercomputer, June 2016).

To alleviate the problem of slow computational speed of MRPrimer, we propose a GPU-based pipeline running on a single machine for primer design, called *GPrimer*, that takes the same input and returns the same output with MRPrimer.

MRPrimer consists of a total of seven MapReduce steps, among which two steps are very time-consuming due to a large amount of computation. GPrimer significantly improves the speed of those two steps by exploiting the computational power of GPUs. Processing the remaining steps using GPUs may rather degrade the performance due to data communication overhead between main memory and GPUs, and thus, GPrimer processes those steps only using CPUs. For exploiting GPUs, GPrimer designs data structures for both (1) coalesced memory access in GPU and (2) workload balancing among GPU threads and (3) copies the data structures between main memory and GPU memory in a streaming fashion for hiding data communication overhead. For human RefSeq DB, GPrimer achieves a speedup of 57 times for the entire steps and a speedup of 557 times for the most time-consuming step using a single machine of 4 GPUs, compared with MRPrimer running on a cluster of six machines. There are many GPU-based methods for sequence data analysis including GPU-BLAST [20], G-BLASTN [21], H-BLAST [22], SOAP3-dp [23], sBWT [24], gCUP [25], Arioc [26], YAMDA [27], and NVIDIA Parabricks [28]. But, to the best of our knowledge, there is no GPU-based pipeline (or method) for primer design, and GPrimer is the first one.

In this paper, we briefly review MRPrimer and present the overview of our GPrimer about how it can improve MRPrimer. Then, we present the details about algorithms and data structures to exploit the computational power of GPUs for the two time-consuming steps. We evaluate the performance of GPrimer compared with MRPrimer and conduct a few experiments for the breakdown analysis of GPrimer. Finally, we draw conclusions.

### Review of MRPrimer

**Fig. 1:**
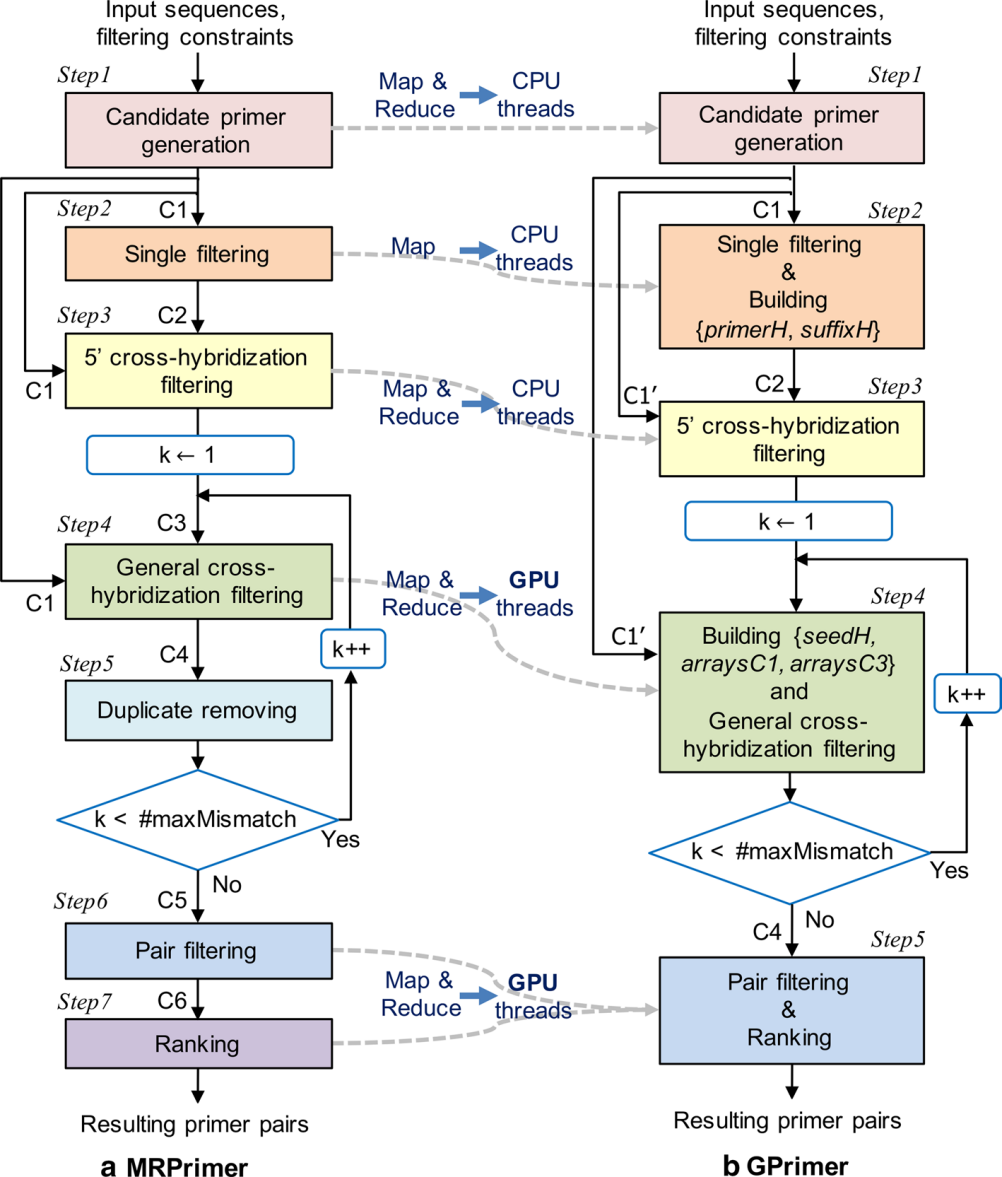
The pipelines of MRPrimer and GPrimer

MRPrimer [5] is a MapReduce-based [29] pipeline that consists of seven steps (Fig. 1a). It takes a sequence DB and a set of filtering constraints as input, and then, after seven steps, returns all feasible and valid primer pairs that exist in the DB.

Step 1 (candidate primer generation) extracts all possible subsequences of the lengths between the minimum length and the maximum length from each sequence, as candidate primers. The lengths are specified by users (e.g., 19–23 bp) as inputs. This step also extracts their reverse complementary primers while tagging them with a symbol ‘*’. The candidate primers generated in this step are used not only for Step 2 (i.e., other single filtering) but also for Steps 3 and 4 (i.e., approximate string matching). Thus, this step just generates all possible subsequences without other single filtering. Step 2 (single filtering) applies the single-filtering constraints to each primer passed from Step 1 and filters out the primers that violate any filtering constraint. The constraints include melting temperature, GC content, self-complementarity, 3’ end self-complementarity, contiguous residue, and Gibbs free energy, which are specified by users as inputs. Step 3 (5’ cross-hybridization filtering) eliminates a candidate primer that is the same as any subsequence of an off-target sequence at the 3’ end and has only a few mismatches (up to four mismatches) at the 5’ end, and so, might cross-hybridize with the off-target sequence due to the high similarity between them, especially at the 3’ end.

Step 4 (general cross-hybridization filtering) eliminates a candidate primer that is similar with any subsequence of an off-target sequence. This step takes two primer sets, *C1* (output of Step 1) and *C3* (output of Step 3). We denote the number of mismatched residues between two primers as *k*. For more efficient computation, this step splits each primer into a set of smaller disjoint pieces (called *seeds*). According to the theorem in [30, 31], and [32], a primer of length $|\textit{P}|$ with at most *k* mismatches must contain a seed exactly matched of at least $\lfloor |P| / (k+1) \rfloor$ residues [1, 5, 8]. All pairs of primers from *C1* and *C3* having a common seed are collected through the shuffle step of MapReduce and checked whether a pair of primers is identical except for *k* residues in the reduce step of MapReduce. After Step 4, there still might be false-positive primers violating the general cross-hybridization filtering constraint. This inherently occurs due to the distributed computation of MapReduce. In order to filter out such primers completely, Step 5 (duplicate removing) rearranges the result of Step 4 in terms of primer and eliminates the primers that do not pass Step 4 in terms of any seeds. The series of Steps 4 and 5 is performed repeatedly while increasing *k* from 1 to the maximum number of mismatch residues (i.e., *#maxMismatch*), which is usually set to 2 [1, 5, 8].

Step 6 (pair filtering) rearranges the result of Step 5 to a set of groups of primers, where each group consists of the primers extracted from the same set of input sequences. Then, it splits the primers of each group into two sets, forward primers and reverse primers, using tags addressed in Step 1, and performs a self-join between them, which applies the pair-filtering constraints to each primer pair. The constraints include length difference, melting temperature difference, product size, pair-complementarity, and 3’-end pair-complementarity, which are specified by users as inputs. The primer pairs passed from Step 6 might not be equally effective even if they satisfy all the given constraints and pass homology tests. Thus, Step 7 (ranking) determines their ranking by calculating a penalty score for each primer pair. The calculation of penalty scores follows the method of Primer3Plus [6].

## Implementation

### Overview of GPrimer

GPrimer is designed to perform the same task with MRPrimer and so return the same result with MRPrimer. But, different from MRPrimer relying on distributed computation, GPrimer exploits GPU computation to handle a large-scale computation required for primer design. Basically, MRPrimer groups a set of values having the same key through the map and shuffle steps of MapReduce and performs a certain function against the set of values in the reduce step of MapReduce, which are done by a lot of parallel processes having their own main memory spaces (i.e., heaps). In contrast, GPrimer groups the set of values using hash maps and performs the function using a lot of CPU or GPU threads within the same main memory space.

GPrimer performs a total of five steps as in Fig. 1b, where Step 4 of GPrimer corresponds to Steps 4 and 5 of MRPrimer, and Step 5 of GPrimer corresponds to Steps 6 and 7 of MRPrimer. Since GPrimer runs in the same memory space, Step 5 of MRPrimer for removing duplicates is not necessary, and also, Step 7 of MRPrimer (i.e., scoring and output formatting) can be performed together with Step 6 of MRPrimer (i.e., pair filtering) as a single step (i.e., Step 5) in GPrimer. Since the general cross-hybridization filtering and pair filtering are the most time-consuming steps, GPrimer performs the Steps 4 and 5 using GPU threads and the remaining Steps 1, 2 and 3 using CPU threads. Performing the Steps 1, 2 and 3 using GPU threads does not improve the performance much due to the overhead of copying data back and forth between main memory and GPU memory.

### Steps 1–3: building hash maps and processing using CPU threads

**Fig. 2:**
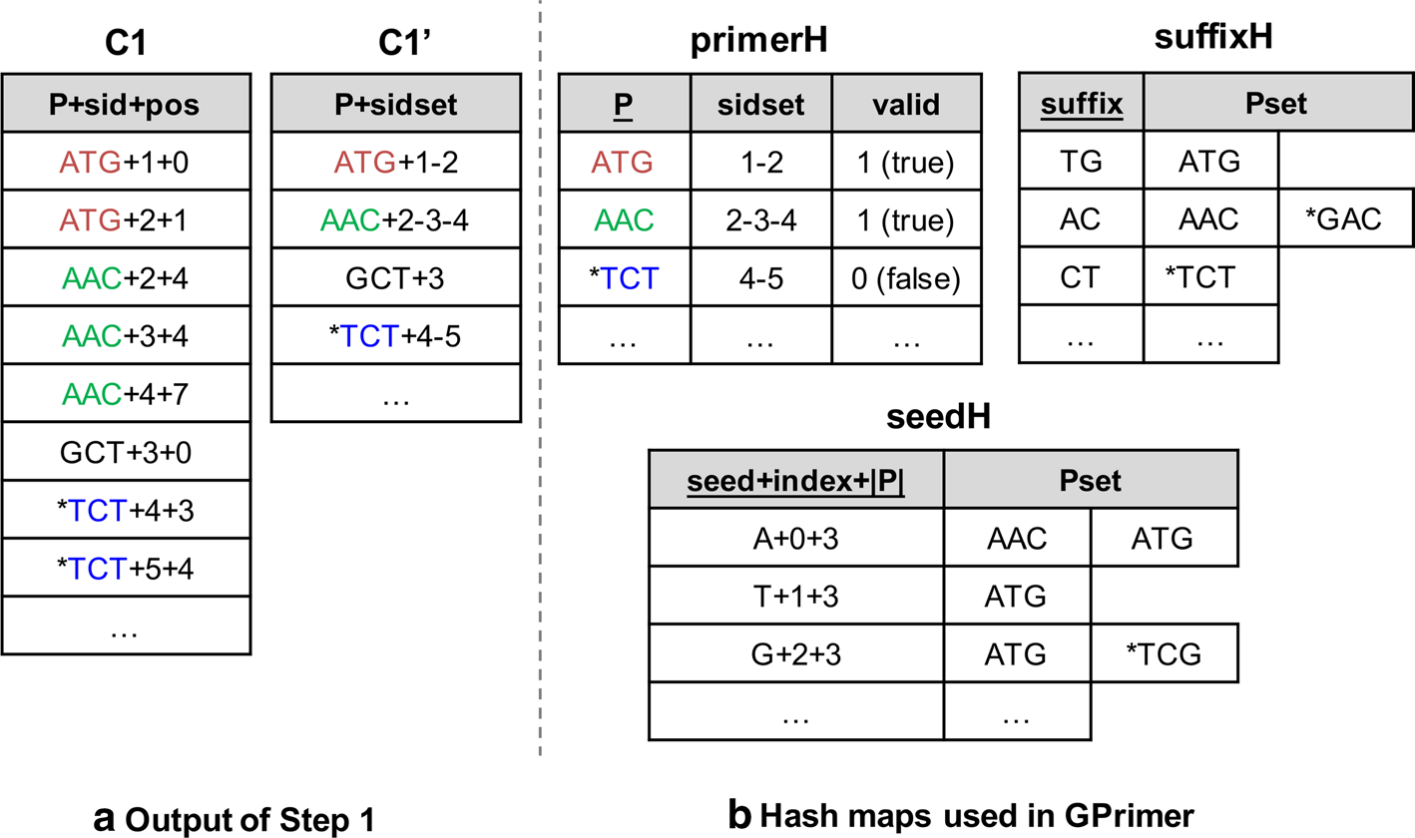
Examples of output of Step 1 and hash maps. **a** Example output of Step 1. **b** Example hash maps used in GPrimer

Step 1 extracts all possible subsequences from a sequence DB for candidate primers as MRPrimer does. We denote the output of Step 1 as *C1*, the output of Step 2 as *C2*, and so on. Figure 2a shows an example of *C1*, where *P* is a primer, *sid* is the ID of the sequence where *P* occurs, and *pos* is the position where *P* occurs in the sequence *sid*. Each row in *C1* is just a concatenation of *P*, *sid*, and *pos*. The symbol ‘+’ is a concatenation operation, and we just use a character ‘+’ for the operation in this paper. Figure 2a also shows an example of *C1’*, which *P* is a primer, and *sidset* is the set of IDs of the sequences where *P* occurs. The set of IDs are concatenated using another character ‘-’. *C1’* is generated together with *C1* as the outputs of Step 1 by grouping a set of rows of *C1* having the same *P* and removing *pos*. *C1* is used for the input of Step 2, while *C1’* is used for the inputs of Steps 3 and 4 for cross-hybridization filtering.

Step 2 applies six filtering constraints to each candidate primer in *C1*. For the candidate primers satisfying the filtering constraints, we build two hash maps, *primerH* and *suffixH*. Figure 2b shows the examples of both hash maps. The former hash map, *primerH* has a primer *P* as a key and a pair of *sidset* where *P* occurs and *valid* as a value, where *valid* indicates whether *P* is valid or not, and all *valid* values in *primerH* are initialized as 1 (i.e., *true*). This hash map is used in Steps 3 and 4. At each step, the *valid* of the primer that does not pass becomes 0. The latter hash map, *suffixH* has a suffix of a primer (i.e., *suffix*) as a key and a set of primers (i.e., *Pset*), where *suffix* occurs, as a value. This hash map is only used in Steps 3. In addition two hash maps, the set of rows of *C1* passing Step 2 is stored as *C2*, which is used in Step 4.

Step 3 filters out the candidate primers which have a common suffix, but different *sidset* with any other primers. It can be done by performing a binary join between *C1’* and *primerH*, where *C1’* contains all possible subsequences in the DB. In detail, we perform the binary join by looking up the hash maps *suffixH* and *primerH* while reading each row of *C1’*. For instance, we assume that we have read a row GCT+3 from *C1’* in Fig. 2. We also assume that there is no primer GCT in *primerH* and *suffixH* since it has been filtered out in Step 2. The primer has a suffix CT, and so, we look up the suffix in *suffixH* and find a set of primers {*TCT}. By looking up the primer *TCT in *primerH*, we find the primer has 4-5 as *sidset*, which is different from the *sidset* of GCT, i.e., 3. That means the primer *TCT may amplify not only the sequences $\{4,5\}$ but also the sequence 3 in wet experiments. Thus, we set *valid* of *TCT to 0 (false) in *primerH*. When we read the next row *TCT+4-5 from *C1’*, we know that the primer *TCT is not valid by looking up *primerH* and so skip looking up *suffixH*. In terms of implementation, the table *C1’* is divided into multiple subtables, and multiple CPU threads read their own subtable and update *primerH*, where there is no race condition since we use atomic operations for looking up and setting *valid* of *primerH*.

### Step 4: building arrays and general cross-hybridization filtering

Step 4 consists of the following two phases: preparing data structures and performing general cross-hybridization filtering. In the first phase, it builds (1) a hash map called *seedH*, (2) a set of arrays built from the result of Step 3 (shortly, *arraysC3*), and (3) a set of arrays built from the result of Step 1 (shortly, *arraysC1*). In the second phase, it performs general cross-hybridization filtering using *seedH*, *arraysC3*, and *arraysC1*, while increasing *k* from 1 to *#maxMismatch*.

The hash map *seedH* is built from the set of valid rows (i.e., *valid*=1) in *primerH* by extracting all possible seeds from *P*. In Fig. 2b, *seedH* shows an example of the hash map when $k=1$, and so, the length of seed is $\lfloor 3 / 2 \rfloor = 1$. The key of *seedH* is a concatenation of *seed*, *index* (position where *seed* occurs in *P*), and |*P*| (length of *P*). The value of *seedH*, i.e., *Pset* contains a set of primers of length |*P*| where *seed* occurs at *index*. For example, in the first row of *seedH* means that the set of primers of length 3 where a seed *A* occurs at the position 0 is {AAC, ATG}. Each set of primers having the same key is grouped in this way. For instance, for human RefSeq DB, a total of 3,309,154 groups are formed when $k=1$, and a total of 77,418 groups are formed when $k=2$. *seedH* is mainly used for building *arraysC3* and *arraysC1*.

**Fig. 3:**
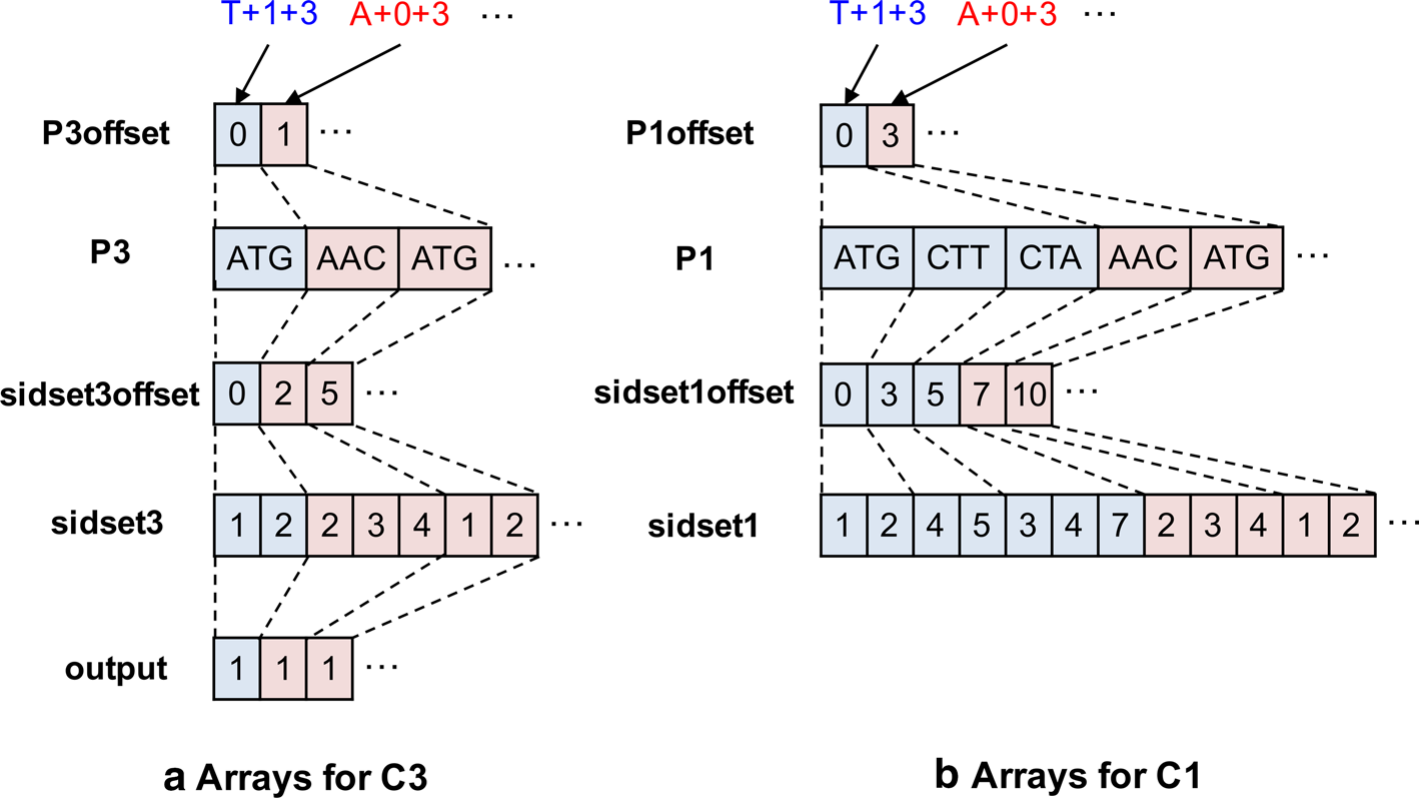
Example of the arrays used in Step 4. **a** *arraysC3*: the set of arrays built from the result of Step 3. **b** *arraysC1*: the set of arrays built from the result of Step 1

The set of arrays, *arraysC3*, is composed of four arrays, *P3offset*, *P3*, *sidset3offset*, and *sidset3* as shown in Fig. 3a. To build *arraysC3*, we first sort the keys of *seedH* in the ascending order of the length of *Pset*, i.e., the number of primers. The order of the keys is used for handling workload balancing of GPU computation, and we call the order as the *workload order*. Then, we build the arrays *P3* and *sidset3* according to the workload order. For example, if we assume that T+1+3 has the shortest *Pset*, and A+0+3 has the second shortest *Pset* in the hash map *seedH*, then *P3* starts with *Pset* of T+1+3 and next *Pset* of A+0+3. Likewise, *sidset3* starts with *sidset* of T+1+3 in *primerH* (i.e., 1-2) and next *sidset* of A+0+3 in *primerH* (i.e., 2-3-4). Both *P3offset* and *sidset3offset* are just pointer arrays for *P3* and *sidset3*, respectively, which is used for coalesced memory access in GPU computation.

The array *output* is for storing the result of general cross-hybridization filtering, whose length is the same with *P3* since the target of filtering is each primer. It is initialized as 1 which means valid and updated during general cross-hybridization filtering. In general, there are multiple elements in *output* for the same primer (e.g., at least three elements for *ATG*).

The set of arrays, *arraysC1*, is composed of four arrays, *P1offset*, *P1*, *sidset1offset*, and *sidset1* as shown in Fig. 3b. Both *P1* and *sidset1* arrays are also built in the same workload order mentioned above. Following the same order is for performing a binary join between *arraysC3* and *arraysC1* quickly and massively using GPU computation. *P1* and *sidset1* are built using *P* and *sidset* in *C1’* (output of Step 1), respectively, while *P3* and *sidset3* are built using *Pset* in *seedH* and *sidset* in *primerH*, respectively. In detail, we extract a set of pairs of key (i.e., *seed+index+*|*P*|) and value (i.e., *Pset*) from each row in *C1’*, if the corresponding key exists in *seedH*, and build *P1* and *sidset1* so as to follow the workload order (e.g., the first T+1+3 and the second A+0+3). The information in *arraysC1* becomes to include that in *arraysC3* since the former is built using the result of Step 1, while the latter is built using the result of Step 3.

All the data structures, *seedH*, *arraysC3* and *arraysC1* need to be constructed for each *k* in Fig. 1b. That is, GPrimer performs the first general cross-hybridization filtering using the data structures constructed for $k=1$ and then the second filtering using the data structures constructed for $k=2$. After the first filtering using $k=1$, *primerH* is updated based on *output* such that, if an element in *output* for a primer is 0, *valid* of the corresponding primer in *primerH* is also set to 0 (false). A new *seedH* should be constructed for $k=2$ since the length of a seed in *seedH* becomes shorter as *k* increases. Accordingly, *arraysC3* and *arraysC1* should be newly constructed based on the new *seedH* and the updated *primerH*. The *output* array also should be initialized.

If the size of input sequence DB is small enough to be processed only with a single GPU, the procedure of Step 4 is relatively simple: preparing data structures in main memory, copying both *arraysC3* and *arraysC1* to GPU memory, and executing a GPU kernel function for general cross-hybridization filtering. However, if the size of DB is too large to be processed even with multiple GPUs at a time, the procedure needs to be more complicated. GPrimer exploits the streaming processing functionality of GPU to make Step 4 scalable in terms of the size of DB and the number of GPUs.

**Fig. 4:**
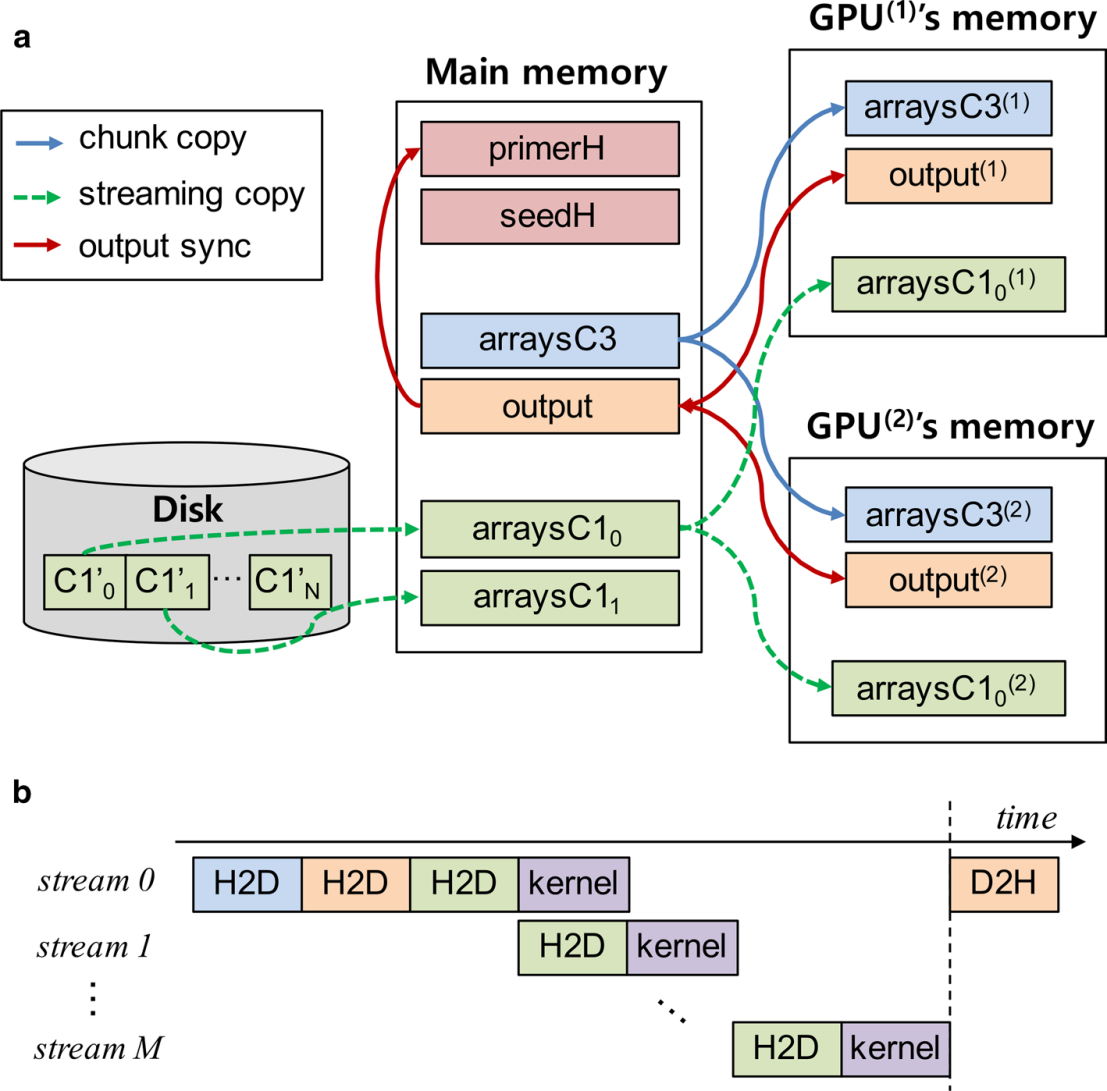
Data flow and tileline in Step 4. **a** Data flow among disk, main memory, and GPUs. **b** Timeline of multiple GPU streams in terms of threee types of GPU operations: H2D(Host to Device) copy, kernel execution, and D2H(Device to Host) copy

Figure 4a shows the flow of data structures of GPrimer among disk, main memory, and GPUs. Basically, GPrimer splits the whole *arraysC3* into multiple disjoint chunks and copies each chunk to each GPU (called chunk-copy). We assume that the number of chunks is equal to the number of GPUs (denoted as *Q*). We denote the chunk assigned to the *y*-th GPU (simply GPU$^{(y)}$) as *arraysC3*$^{(y)}$. In Fig. 3a, each key (i.e., *seed+index+*|*P*|) and its corresponding subarrays in *P3offset*, *P3*, *sidset3offset*, and *sidset3* is the smallest unit of independent workload. Thus, *arraysC3* is split at the boundary between keys into multiple chunks which are almost equal in size. GPrimer splits *output* into chunks and copies each chunk to each GPU in a similar way.

In case of *arraysC1*, the table *C1’* is divided into multiple subtables *C1’*$_x$, and we denote the part of *arraysC1* built from *C1’*$_x$ so as to follow the workload order as *arraysC1*$_{x}$. We can again split each *arraysC1*$_{x}$ into multiple disjoint chunks *arraysC1*$_{x}^{(y)}$ such that the range of keys of *arraysC1*$_{x}^{(y)}$ is the same to that of *arraysC3*$^{(y)}$. GPrimer copies *arraysC1*$_{0}^{(y)}$ to GPU$^{(y)}$ ($0 \le y < Q$), copies *arraysC1*$_{1}^{(y)}$ to GPU$^{(y)}$ ($0 \le y < Q$) and so on (called streaming-copy).

Here, streaming-copy means that the GPU kernel function for a pair of *arraysC3*$^{(y)}$ and *arraysC1*$_{x}^{(y)}$ is executed in a GPU simultaneously during *arraysC1*$_{z}^{(y)}$ is prepared and copied to the GPU ($x < z$). For streaming-copy, we need to use multiple GPU streams, where a GPU stream means an ordered sequence of the following three types of operations: H2D copy, kernel execution, and D2H copy [33]. Figure 4b shows the timeline of multiple GPU streams in GPU$^{(y)}$. The green H2D boxes correspond to streaming-copying *arraysC1*$_{x}^{(y)}$ to GPU and the purple boxes executing the GPU kernel function. Once the kernel function has processed *arraysC1*$_{x}^{(y)}$, its result (i.e., validity of primers) is in *output*$^{(y)}$ in GPU memory. Thus, we need to synchronize *output* by copying *output*$^{(y)}$ from GPU$^{(y)}$ to main memory while merging them (orange D2H box). After completing Step 4 for a specific *k*, *primerH* is updated based on *output* in main memory. We have assumed so far that the whole *arraysC3* can fit in the memory of multiple GPUs, and it actually is for all input DBs we have tested. If it does not fit in, GPrimer divides *arraysC3* into multiple parts such that each part can fit in and repeats the above procedure like a nested loop method.

Algorithm 1 presents the pseudo code of the GPU kernel function for Step 4, which may seem complicated, but actually is simple. The function mainly relies on coalesced memory access in GPU memory for efficiency, and so, most of its lines are about identifying memory addresses to be accessed. In the function, the basic processing unit is a GPU thread, and the basic data unit to be processed is a key (i.e., *seed+index+*|*P*|). For simplicity, we denote *arraysC3*$^{(y)}$, *arraysC1*$_{x}^{(y)}$, and *output*$^{(y)}$, as *arraysC3*, *arraysC1*, and *output*, respectively. Both arrays, *arraysC3* and *arraysC1*, can be divided by each key. For example, in Fig. 2a, the blue part corresponds to the key $T+1+3$, and the red part to the key $A+0+3$. The function takes *workloadThreshold* and *k* as inputs and updates and returns the *output* array as output. The parameter *workloadThreshold* is the boundary that determines whether each thread processes a single key (*each-thread* mode), or all threads of a thread block process a single key (*block-threads* mode). The set of keys having smaller index than *workloadThreshold* (e.g., the index of $A+0+3$ is 1 in Fig. 2a) is processed in the each-thread mode using Algorithm 1. We will explain how to determine *workloadThreshold* later in more detail. 
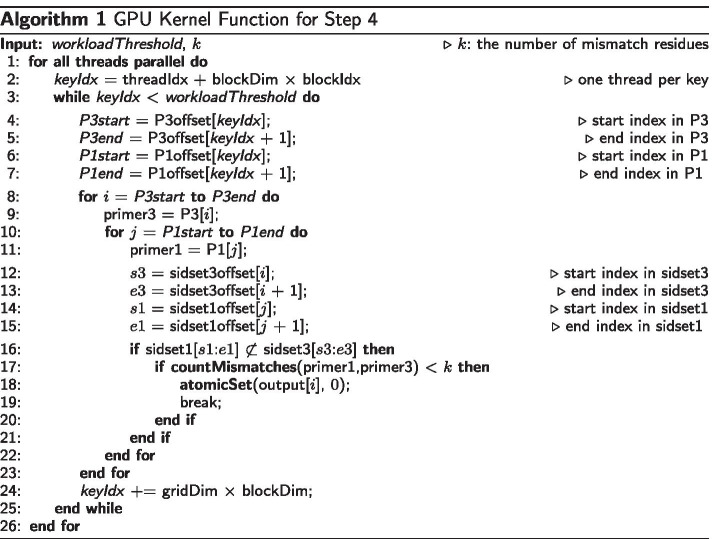


Since it is a GPU kernel function, all GPU threads execute the same entire function (Line 1). At Line 2, each thread gets the index of a key that it processes based on its own index (i.e., threadIdx), the size of a thread block (i.e., blockDim) and the index of the block (i.e., blockIdx). After a thread processes its own key (Lines 4–23), it gets the index of the next key (Line 24) and repeats processing the key. The thread gets the start and end indices in *P3* (Lines 4–5) and the start and end indices of in *P1* (Lines 6–7). Then, it checks every pairs of primers between *P*3[*P*3*start* : *P*3*end*] and *P*1[*P*1*start* : *P*1*end*] (Lines 8–11). For a specific pair of *primer3* and *primer1*, it gets the start and end indices in *sidset3offset* (Lines 12–13) and the start and end indices of in *sidset1offset* (Lines 14–15). Then, it sets *output*[*i*] for *primer3* to 0, i.e., invalid, (Line 18), if *primer3* is similar to *primer1* with up to *k* mismatch residues (Line 17), and at the same time, *primer1* amplifies any target sequences that *primer3* does not amplify (Line 16). The logic in Lines 16–19 has been presented in the previous studies [1, 5, 8].

Table 1 shows the amount of computation of Step 4 in terms of the number of pairs of *primer3* and *primer1* to be processed, i.e., the number of executions of Line 12–21 in Algorithm 1. It exceeds 1.4 trillions for human and mouse, which indicates Step 4 is indeed compute-intensive.

**Table 1:** The amount of computation of Step 4 for six species.

	k=1	k=2	Sum
Pig	310,393,843	21,607,890,291	21,918,284,134
Cow	4,457,131,080	313,019,951,055	317,477,082,135
Zebrafish	6,835,870,424	435,727,302,462	442,563,172,886
Rat	9,058,344,862	625,330,936,147	634,389,281,009
Mouse	21,172,185,165	1,458,103,349,355	1,479,275,534,520
Human	19,851,609,914	1,392,486,084,329	1,412,337,694,243

Now, we explain about the parameter *workloadThreshold*, which is the boundary that determines whether each thread processes a single key (*each-thread* mode), or all threads of a thread block process a single key (*block-threads* mode). The set of keys having smaller index than *workloadThreshold* (e.g., the index of $$A+0+3$$ is 1 in Fig. 3a) is processed in the each-thread mode. The distribution of the numbers of primers per key in the array *P3* (or *P1*) is highly skewed. For instance, the minimum number of primers per key is 1, while the maximum number is 8786 for human RefSeq DB ($$k=2)$$. That means some GPU threads process a few keys, while some other GPU threads process thousands keys. The former threads do nothing until the latter threads complete the execution of the function, which may severely reduce the utilization of GPU computation and so degrade the performance of Step 4. The hybrid approach that uses either the each-thread mode or the block-threads mode depending on the workload (i.e., the number of primers per key) can alleviate this problem. Since *P3* is already sorted by workload, and *P1* is also almost sorted by workload, we can just perform the each-thread mode for the keys having smaller indices than *workloadThreshold* and the block-threads mode for the other keys. In the block-threads mode, we usually set the number of blocks to 1024 and the size of a block to 1024 threads. That is, up to 1024 keys are processed in parallel using 1024 threads per key.

The number of primers per key tends to be increased as *k* increases since the number of unique seeds is decreased as *k* increases. Thus, we set *workloadThreshold* to a smaller percentile when $k=2$ compared to when $k=1$. We heuristically set *workloadThreshold* to the 85th percentile for $k=1$, while setting it to the 20th percentile for $k=2$. That is, if there are a total 100 keys, *workloadThreshold* becomes 85 for $k=1$. For human RefSeq DB, the number of primers of the key of the 85th percentile is 22 ($k=1$), and that of the 20th percentile is 164 ($k=2$). That means the keys having up to 22 and 164 primers are processed using a single thread when $k=1$ and $k=2$, respectively.

### Step 5: pair filtering and ranking

**Fig. 5:**
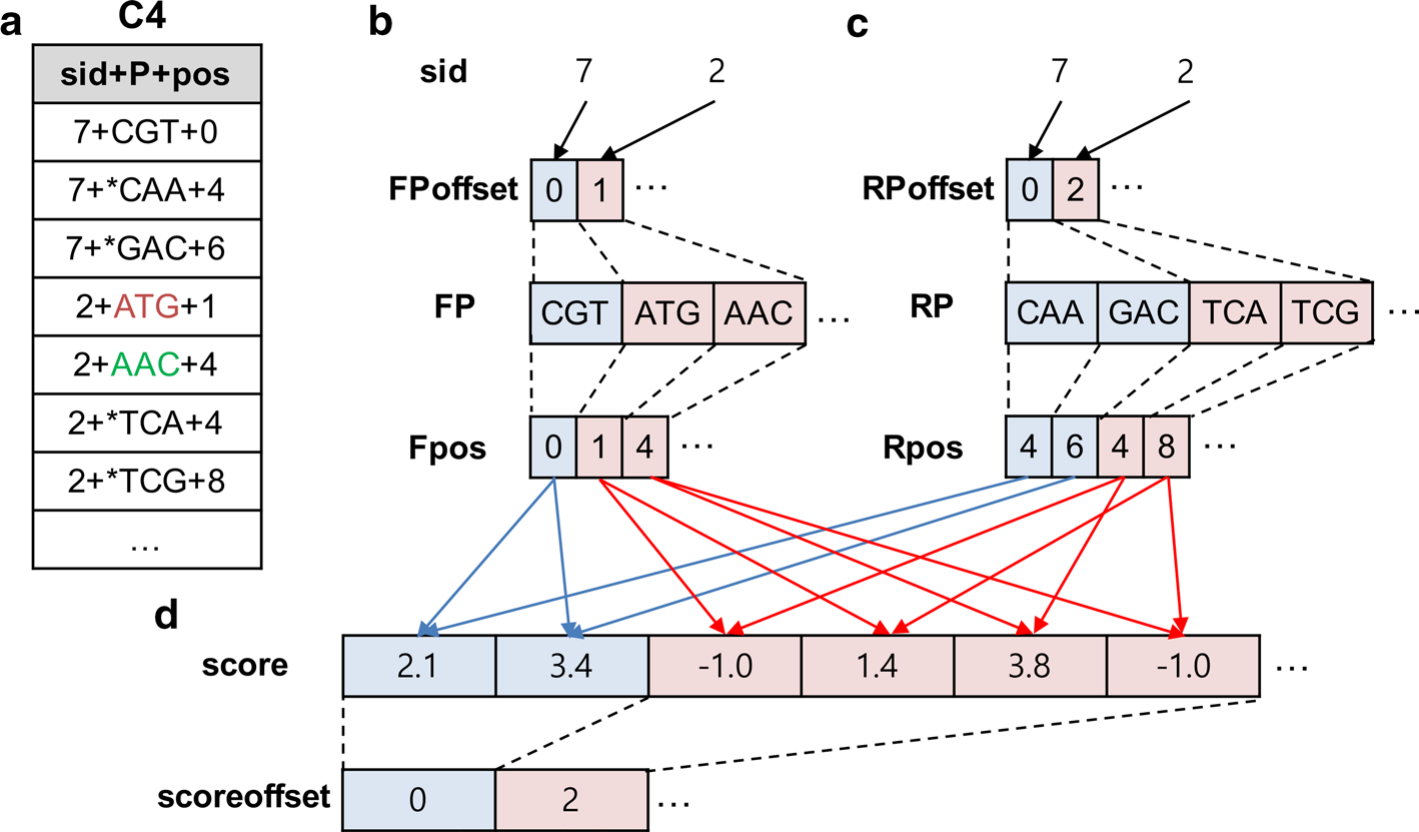
Example of data structures used in Step 5. **a** Sorted *C4*. **b** Arrays for forward primers. **c** Arrays for reverse primers. **d** Arrays for the scores of pairs of primers

GPrimer processes Step 5 as well as Step 4 by exploiting coalesced memory access using arrays, hiding PCI-E communication overhead using multiple asynchronous GPU streams, and load balancing using *workloadThreshold*. At the end of Step 4, GPrimer simply reads each row of *C2* and writes the row into *C4* if its *valid* is 1 in *primerH*. Step 5 takes the result of Step 4, i.e., *C4*, as input, and groups it by *sid*. For constructing arrays, GPrimer counts the number of forward (or reverse) primers for each *sid* group in *C4* and sorts the groups in *C4* in the ascending order of the number of primers per group (i.e., workload order). Figure 5a shows an example of *C4* sorted by the workload order.

GPrimer constructs two kinds of arrays, one for forward primers and the other for reverse primers, using *C4* and applies five pair filtering constraints to all possible pairs between forward primers (FPs) and reverse primers (RPs). Then, for all valid pairs of FP and RP satisfying the constraints, GPrimer calculates their scores, sorts the pairs by their scores, and stores the formatted result of the pairs as output. In detail, it constructs three arrays, *FPoffset*, *FP* and *Fpos*, for forward primers, and three arrays, *RPoffset*, *RP* and *Rpos*, for reverse primers, according to the workload order. For example, *FP* starts with CGT for the group sid:7 and next {ATG, AAC} for the group sid:2 in Fig. 5b. Likewise, *RP* starts with {CAA, GAC} for sid:7 and next {TCA, TCG} for sid:2 in Fig. 5c. GPrimer also prepares the array called *score* for storing the scores of all possible pairs between FPs and RPs (Fig. 5d). By scanning both *FPoffset* and *RPoffset* once, we can construct *scoreoffset*, i.e., the pointer array for *score*. It is initialized with $-1.0$, which means the corresponding pair is invalid. If a pair of primers is valid, i.e., satisfies the pair filtering constraints, its score is calculated and assigned to the corresponding element in the array *score*.

**Fig. 6:**
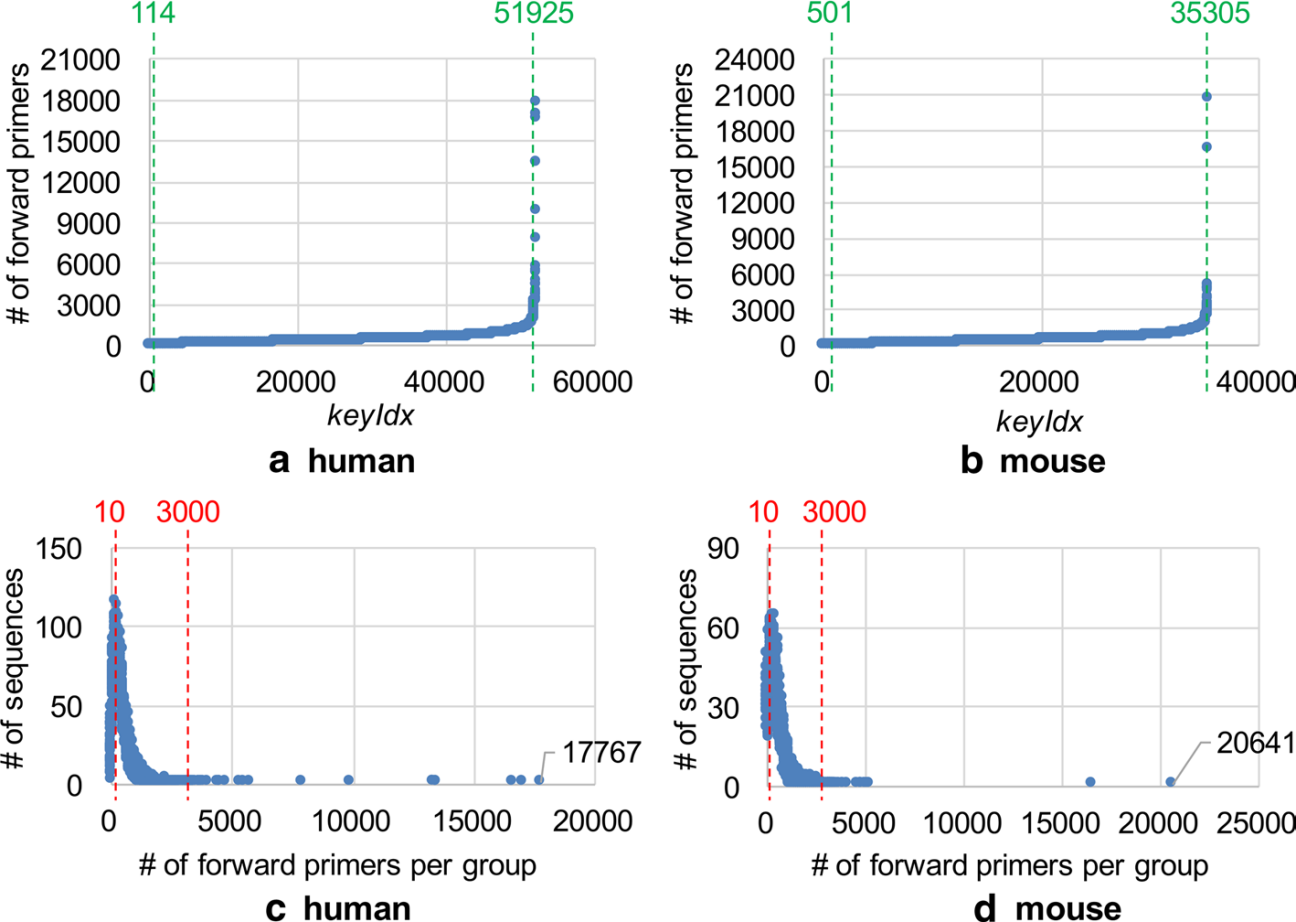
Distribution for workload in Step 5. **a** and **b** represent the distributions of workload per group for human and mouse RefSeq DBs, respectively. **c** and **d** represent the distributions of the number of sequences per workload for human and mouse RefSeq DBs

The GPU kernel function for Step 5 processes pairs of primers between FP and RP. For instance, in Fig. 5b–d, the first GPU thread (*keyIdx*=0) processes two pairs of primers in blue, and the second GPU thread (*keyIdx=1*) processes four pairs of primers in red. The exact workload should be the number of primer pairs, but we regard the number of forward primers as the amount of workload for simplicity. The distribution of the workload in Step 5 is more highly skewed than that in Step 4. Figure 6a, b show the distributions of workload per group for human and mouse, where *x*-axis is *keyIdx* (i.e., group), and *y*-axis is the number of forward primers per group (i.e., workload). The figures indicate that a lot of sequences (groups) have relatively small workload, while a few sequences have very large workload. Figure 6c, d show the detailed distributions for human and mouse, where *x*-axis is workload, and *y*-axis is the number of sequences per workload. Based on this observation, we use two workload thresholds,*smallThreshold* and *largeThreshold* for Step 5, instead of a single threshold used in Step 4. We perform the each-thread mode for the groups having smaller indices than *smallThreshold* and the block-threads mode for the groups having the indices between *smallThreshold* and *largeThreshold*. For the groups having larger indices than *largeThreshold*, we make all the threads of a GPU process a single group (*all-threads* mode). We usually set the number of blocks to 1024 and the size of a block to 512 threads for both the block-threads and all-threads modes. For human, the groups of *keyIdx*[0:114] are processed in the each-thread mode, the groups of *keyIdx*[114:51925] in the block-threads mode, and the groups of *keyIdx*[51925:] in the all-threads mode. The last group contains 17767 forward primers and 17713 reverse primers, and a total of more than 300 million pairs of the primers are processed using 512 thousand threads in the all-threads mode.

In Step 4, GPrimer streaming-copies *arraysC1* to GPUs if it does not fit in GPU memory. Likewise, in Step 5, GPrimer streaming-copies six arrays for FPs and RPs and two arrays for scores to GPUs if they do not fit in GPU memory. Since each group is an independent workload in Step 5, GPrimer streaming-copies the arrays for disjoint sets of groups to different GPUs. The resultant *score* array in each GPU is copied back to main memory for synchronization. Ranking (i.e., sorting) of the pairs by their scores is done by CPU threads.

## Result

### Experimental setup and data sets

MRPrimer [5] was evaluated using MapReduce on a cluster of six server machines: one master and five slaves. Each machine is equipped with two Intel Xeon 10-core CPUs, 512 GB main memory, and 6 TB disk. We used 40 Map processes and 40 Reduce processes per machine (i.e., a total of 200 Map and 200 Reduce processes). GPrimer was evaluated using a single machine equipped with the same CPUs, the same main memory, but eight NVIDIA GTX 1080 ti GPUs having 11 GB device memory.

**Table 2:** The size of data in each step (input: the number of sequences, *C1*–*C4*: the number of rows, output: the number of pairs of primers)

	Pig	Cow	Zebrafish	Rat	Mouse	Human
Input	4,180	13,382	15,876	17,639	35,349	51,979
C1	77M	308M	344M	414M	1119M	1831M
C2	3.1M	11.7M	16.8M	18.9M	48.5M	65.9M
C4	2.8M	9.6M	13.3M	14.7M	35.1M	47.5M
Output	21M	64M	111M	107M	234M	278M

For data sets, we used the mRNA sequence DBs for six species—human, mouse, rat, zebrafish, cow, and pig—from the NCBI Reference Sequence (RefSeq) database (http://www.ncbi.nlm.nih.gov/refseq/). The RefSeq DBs used contain a total of 138,375 mRNA sequences that have NM as the prefix of GenBank accession number (the version that updated at 21 November 2018 for human, and 7 November 2018 for others). Table 2 summarizes the size of data in each step. The number of rows in output becomes much larger than that in *C4* since each row is not a single primer, but a pair of primers.

### Performance comparison with MRPrimer

**Fig. 7:**
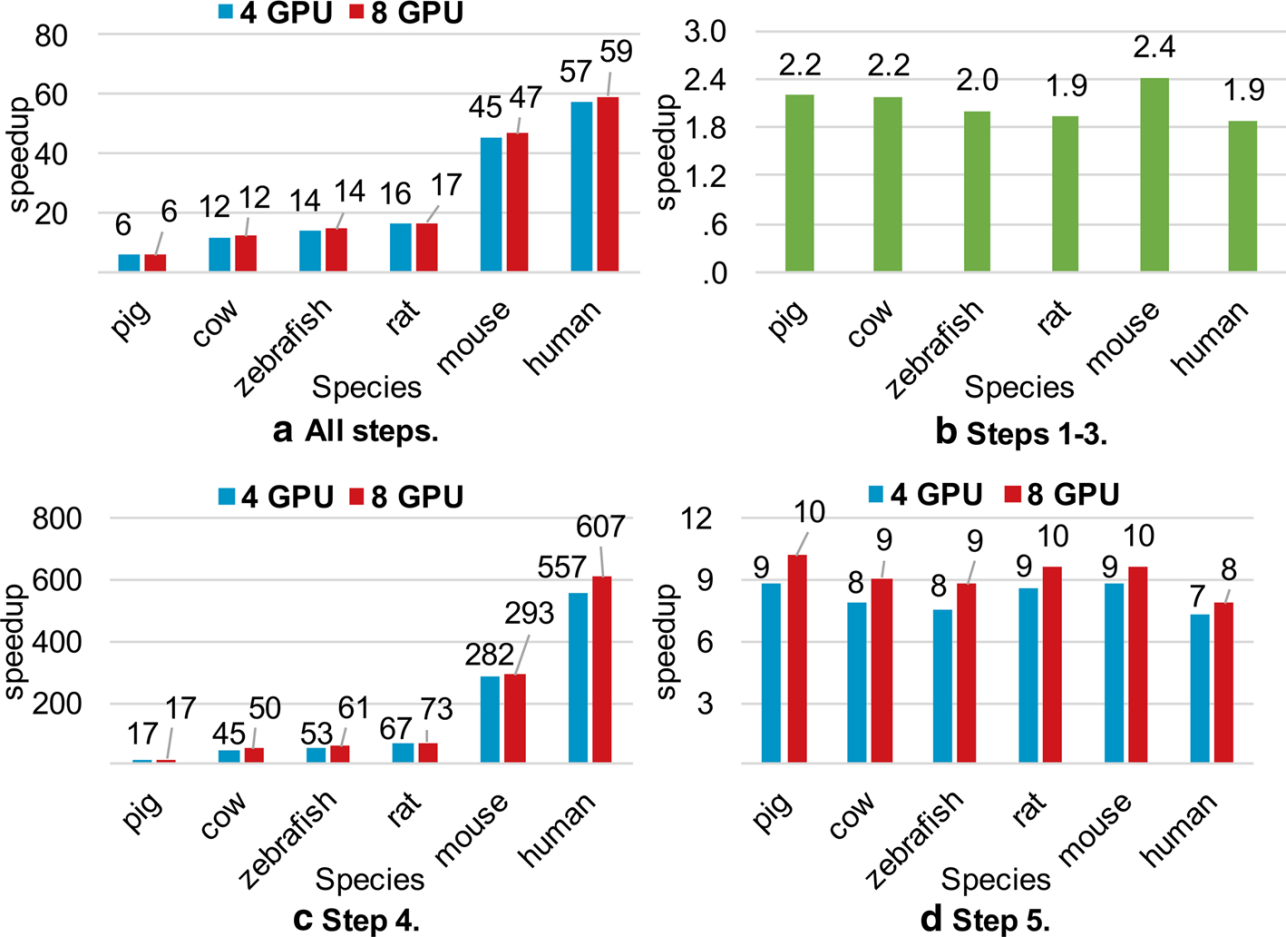
Speedup of GPrimer compared to MRPrimer. **a** speedup for all steps; **b** speedup for Steps 1–3 (not utilizing GPUs); **c** speedup for Step 4; **d** speedup for Step 5

We evaluated the elapsed times of GPrimer and MRPrimer. Figure 7a shows the speedup of GPrimer compared to MRPrimer for all steps for all species. In the figure, the speedup using eight GPUs is higher than that using four GPUs, but the difference is not much, which will be explained in the next section. The speedup also becomes larger as the size of an input sequence DB increases, for example, from pig to human. We can say that it is a desirable property that the speedup is more improved for a larger sequence DB. Figure 7b shows the speedup for Steps 1–3, where GPrimer only uses CPU threads as MRPrimer does. GPrimer improves the performance about twice compared to MRPrimer, even though GPrimer exploits 20 CPU cores, while MRPrimer exploits a total of 100 CPU cores. The performance improvement is mainly due to that the threads in GPrimer compute in the same memory space, while those in MRPrimer compute in different memory spaces with some overhead of network communication. Figure 7c shows the speedup for Step 4, i.e., general cross-hybridization (GCH) filtering step. We performed GCH filtering twice, i.e., for $k = 1$ and $k = 2$, and summed both elapsed times for evaluation. The speedup is quite large, in particular, up to 607 times for human. In terms of elapsed times, for human, GPrimer took about only 11 minutes using four GPUs, while MRPrimer took about 100 h. This indicates that our method for Step 4 described in Implementation is quite effective to exploit the computational power of GPU. Figure 7d shows the speedup for Step 5, i.e., pair filtering and ranking step, where the speedup is about 10. Thus, Step 5 is more compute-intensive compared to Steps 1–3, but much less compute-intensive compared to Step 4.

### Performance of GPrimer varying the number of GPUs

**Fig. 8:**
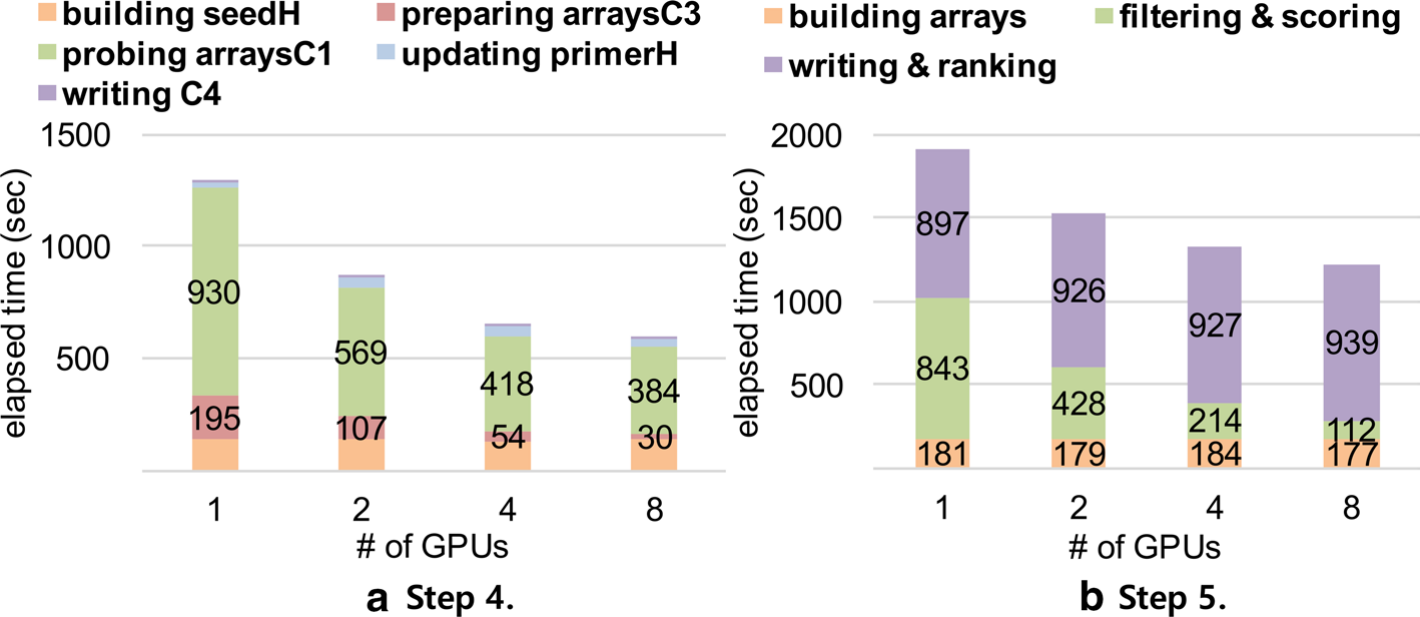
Elapsed times of the substeps in Steps 4 and 5 of GPrimer while varying the number of GPUs for human RefSeq DB. Each bar in graphs indicates the total elapsed time. The probing C1 phase in **a** and filtering and scoring phase in **b** are the phases that perform GPU operations

Figure 8a shows the elapsed times of Step 4 of GPrimer for human while varying the number of used GPUs. Step 4 is carried out in the order of five substeps: building *seedH*, preparing *arraysC3*, probing *arraysC1* against *arraysC3*, updating *primerH*, and writing *C4*. In the figure, the substep of probing *arraysC1* includes not only the time of executing the GPU kernel function, but also the time of building each chunk of *arraysC1* in main memory using CPUs and streaming it to GPU memory. In fact, three different types of operations, i.e., building, streaming, and executing, for *arraysC1* overlap much in the timeline. The time of probing *arraysC1* decreases as the number of GPUs increases, since the substep is executed in GPUs. But, the time is not much decreased for four or eight GPUs, since the overhead of building and streaming *arraysC1* gets larger. The time of only executing the GPU kernel function decreases in inverse proportion to the number of GPUs, as in Table 3. Here, the reason why the time decreases by more than twice sometimes when the number of GPUs becomes twice is that the memory usage for *arraysC3* in each GPU is reduced as the number of GPUs increases, and so we can reduce the number of executions of the GPU kernel function by increasing the size of a chunk of *arraysC1*. The substep of preparing *arraysC3* means building *arraysC3* in main memory and chuck-copying it GPU memory. As the number of GPUs increases, the size of the chunk of *arraysC3* assigned to each GPU decreases, and so, the time of this substep also decreases. The times of other three substeps are almost the same regardless of the number of GPUs.

Figure 8b shows the elapsed times of Step 5 of GPrimer. Step 5 is carried out in the order of three substeps: building arrays, filtering & scoring, and writing & ranking. In the figure, the substep of filtering & scoring is processed by GPUs, and so its time decreases approximately in inverse proportion to the number of GPUs. On the contrary, the elapsed times of the other two substeps are almost the same regardless of the number of GPUs, since they are not related to GPU computation.

**Table 3:** Elapsed times of executing the GPU kernel function for human RefSeq

	1 GPU	2 GPUs	4 GPUs	8 GPUs
Step 4 (k=1)	69 s	26 s	19 s	9 s
Step 4 (k=2)	836 s	452 s	250 s	118 s
Step 5	828 s	415 s	204 s	103 s

### Effectiveness of workload balancing and streaming-copying

**Fig. 9:**
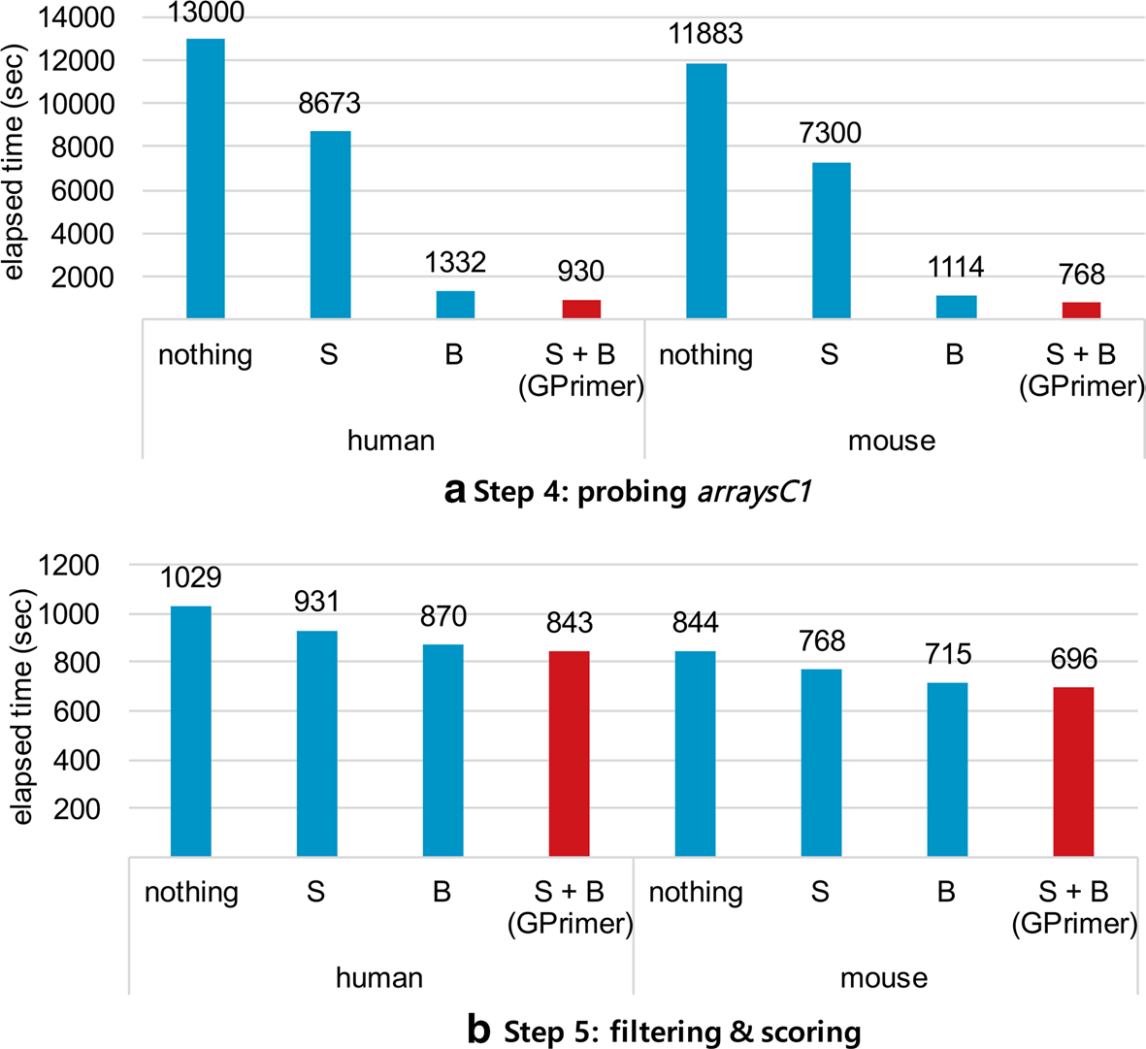
Elapsed times of four different versions of GPrimer while turning on and off two techniques, workload balancing (B) and streaming-copying (S). The experiments are performed for human and mouse RefSeq DBs (indicated in x-axis)

To fully exploit GPUs, GPrimer relies on coalesced memory access within GPU, workload balancing among GPU threads, and streaming-copying data between main memory and GPU memory. Among three techniques, we check the effectiveness of workload balancing and streaming-copying in this section. It is hard to turn on and off coalesced memory access, and so we skip checking its effectiveness. Figure 9 shows the breakdown of performance of GPrimer, in particular, the substeps using GPUs, i.e., probing *arraysC1* in Step 4 and filtering & scoring in Step 5. We evaluated the elapsed times of the following four versions of GPrimer: only using streaming-copying (S), only using workload balancing (B), using neither workload balancing nor streaming-copying (nothing), and using both (S+B). Not using streaming-copying means synchronous copying after completing the execution of the GPU kernel function. Not using workload balancing means processing keys or groups only in a single mode, in particular, only the each-thread mode for Step 4 and only the block-threads mode for Step 5, without considering thresholds. We could not use only the each-thread mode for Step 5 due to lack of GPU memory. In Fig. 9a, we can see workload balancing is more important than streaming-copying for performance. In Fig. 9b, workload balancing and streaming-copying in Step 5 are less effective than those in Step 4. In detail, streaming-copying is less effective since the size of streaming-copied data is smaller, and the data flow of streaming-copying is simpler. Workload balancing is also less effective since the block-threads mode is overall the best single mode among three modes, each-thread mode, block-threads mode, and all-threads mode, in terms of handling the skewness of workload.

### Memory usage

Table 4 shows the memory usage for major data structures in GPrimer (in MByte). The hash maps, *primerH*, *suffixH*, and *seedH*, are in main memory. The remaining data structures all are in GPU memory. As you can see, the size of *arraysC3*+*output* is small enough to fit in GPU memory even for human and mouse, and so streaming *arraysC1* to GPUs needs to be done only once. The biggest data structure is a pair of *score* and *scoreoffset*, but no memory problem occurs since they are allocated, calculated, and copied back to main memory in a small size enough to fit in GPU memory.

**Table 4:** Memory usage for major data structures (MB)

	Mouse		Human	
	$k=1$	$k=2$	$k=1$	$k=2$
primerH	9808		11,664	
suffixH	5530		5125	
seedH	3649	1812	3390	1721
arraysC1	34,308	64,412	40,191	77,200
arraysC3+output	1979	3921	1967	2848
FPoffset+FP+Fpos	1431		1936	
RPoffset+RP+Rpos	1430		1937	
score+scoreoffset	112,026		143,407	

Table 5 shows the memory overhead of MRPrimer and GPrimer for mouse RefSeq DB. Since MRPrimer does not maintain separate major data structures, we have measured the peak main memory usage of both in each step. For MRPrimer, we sum the memory usage of six machines used. We note that the peak memory of GPrimer in Steps 6 and 7 (i.e., about 85 GB) is smaller than the size of the data structures of *score* and *scoreoffset* (i.e., 112 GB) in Table 4. This is because GPrimer does not generate *score* and *scoreoffset* at once, but rather generate them step by step in a smaller size that can fit into GPU memory.

**Table 5:** Peak memory usage of MRPrimer and GPrimer in each step for mouse RefSeq DB (in MB)

	Step 1	Step 2	Step 3	Step 4	Step 5	Step 6	Step 7
MRPrimer	44,527	239,306	214,869	310,615	43,664	35,404	312,558
GPrimer	266	16,240	16,342	77,978		84,972	

## Conclusions

In this paper, we have proposed a fast GPU-based pipeline for primer design called GPrimer that can significantly improve the performance of the existing MRPrimer pipeline based on MapReduce. GPrimer takes the same input data and returns the exactly same output with MRPrimer. That is, GPrimer has the exactly same specificity of primer design with MRPrimer. The only difference between GPrimer and MRPrimer is the speed of primer design. MRPrimer is a MapReduce-based pipeline, but GPrimer is a GPU-based pipeline. Due to the proposed data structures and algorithms in this paper, for human RefSeq DB, GPrimer achieved a speedup of 57 times for the entire steps and a speedup of 557 times for the most time-consuming step, i.e., homology test step, using a single machine of 4 GPUs, compared with MRPrimer running on a cluster of six machines. GPrimer not only significantly outperforms MRPrimer, but also its improvement is more marked as the size of sequence DB increases. Since GPrimer has the exactly same specificity of primer design with MRPrimer, GPrimer also outperforms other primer design software that MRPrimer has outperformed, in terms of specificity. Therefore, we believe GPrimer can be significantly used for web-based primer design tools and primer databases for detecting RNA viruses to deal with the sizes of sequence DBs growing exponentially.

## Availability and requirements

- Project name: GPrimer.
- Project home page: http://github.com/qhtjrmin/GPrimer.
- Operating system: Linux Ubuntu 16.04 LTS or higher.
- Programming language: C++11, CUDA.
- Other requirements: CUDA toolkit version 8 or higher. Nvidia driver (v384 or higher), GCC/G++ 4.8.x or later.
- License: BSD-3-Clause.
- Restrictions to use by non-academics: Not applicable.

## Data Availability

The used input datasets are from the NCBI Reference Sequence (RefSeq) database (http://www.ncbi.nlm.nih.gov/refseq/), and the datasets used in the current study are available in the Github repository, http://github.com/qhtjrmin/GPrimer. The source code for the proposed tool GPrimer is available from the same Github repository under BSD-3-Clause license.

## Abbreviations

PCR: Polymerase chain reaction
DB: Database
CPU: Central processing unit
GPU: Graphical processing unit
CUDA: Compute unified device architecture
